# The existence of Th22, pure Th17 and Th1 cells in CIN and Cervical Cancer along with their frequency variation in different stages of cervical cancer

**DOI:** 10.1186/s12885-015-1767-y

**Published:** 2015-10-16

**Authors:** Wenjing Zhang, Xinli Tian, Fidia Mumtahana, Jun Jiao, Teng Zhang, Kimiko Della Croce, Daoxin Ma, Beihua Kong, Baoxia Cui

**Affiliations:** 1 Department of Obstetrics and Gynecology, Qilu Hospital, Shandong University, Jinan, 250012 P.R. China; 2 Key Laboratory of Gynecologic Oncology, Qilu Hospital, Shandong University, Jinan, 250012 P.R. China; 3 Hematology Oncology Center, Qilu Hospital, Shandong University, Jinan, 250012 P.R. China; 4 Department of Obstetrics and Gynecology, Weifang Maternal and Child Health Hospital, Weifang, 261011 P.R. China; 5 Department of Molecular & Cellular Biology, University of Arizona, Tucson, AZ USA

**Keywords:** Cervical cancer, Th17, Th22, Th1, IL-22

## Abstract

**Background:**

Recently, it is found that T-helper (Th) 22 cells are involved in different types of autoimmune and tumor diseases. But, till now, no study has been carried out to understand the involvement of these cells in cervical cancer (CC).

**Methods:**

Flow cytometry was used to determine the expression of interferon gamma (IFN-γ), Interleukin-22 (IL-22), IL-17 in the peripheral blood of healthy controls (HC), CIN and cervical cancer patients. From peripheral blood mononuclear cells (PBMCs), mRNA expression levels of Aryl hydrocarbon receptor (AHR), RAR-related orphan receptor C (RORC), TNF-α and IL-6 were respectively determined. Using the method of ELISA, plasma concentrations of IL-22, IL-17 and TNF-α were examined.

**Results:**

Th22 and Th17 cells were elevated in CC and CIN patients. Th1 cells and the plasma concentrations of IL-22 in CC patients were significantly increased compared with HC. In CC patients, an increased prevalence of Th22 cells was associated with lymph node metastases. There was a positive correlation between Th22 and Th17 cells, but an approximately negative correlation between Th22 and Th1 cells in CC patients. The mRNA expression of RORC, TNF-α and IL-6 was significantly high in CC patients.

**Conclusions:**

Our results indicate that there is a higher circulatory frequency of Th22, Th17 and Th1 cells in CC which may conjointly participate in the pathogenesis and growth of CC.

## Background

Cervical cancer (CC) is one of the leading gynecological cancers in developing countries. The main etiology behind this occurrence is the persistent infection of high-risk human papillomavirus (HPV) [1–3]. Even if the incidence of HPV is high, with the help of cell mediated immunity, it can be cleared spontaneously [4–6]. A very few cases may develop into advanced CC from precancerous lesions which may indicate a substantial role of immune regulation in the controlling of HPV associated lesions and cancer progression [7].

We know that T helper (Th) cells, one subgroup of lymphocytes, have an essential role in the immune system. Recently it was demonstrated that Th cells such as Th1, Th2, Th17, Treg cells, participate in the pathogenesis and progression of different solid tumors [7–10]. A newly discovered T cell subset - Th22 cells, which were detected in autoimmune and inflammatory diseases, have the ability to secrete IL-22 and TNF-α, but do not express IL-4 (Th2 marker), IL-17 (Th17 marker) or IFN-γ (Th1 marker). In the human body (in the presence of IL-6 or/and TNF-α) the naive CD4^+^cells differentiate into Th22 cells with the aid of plasmacytoid dendritic cells, AHR and RORC [11, 12]. It is known that Th22 is a distinct subset with novel characteristics compared to other Th cells (Th17, Th2 and Th1 cells). It is demonstrated that Th22 cells play an important role in the pathogenesis of inflammatory diseases and autoimmunity diseases such as psoriasis, Graves’ disease and rheumatoid arthritis. [13–15]. However, the nature of Th22 cells are not properly umderstood in human cancer. Recently some studies concluded that Th22 cells contribute to the the progression of hepatocellular and gastric carcinoma which indicates that Th22 cells may be involved in the development of tumors. [16–18].

IL-22 which belongs to the IL-10 cytokine family is mainly an effector cytokine of Th22 cells. IL-22 maintains its function by binding to a heterodimeric transmembrane receptor complex consisting of IL-10R2 and IL-22R1, and activates Janus kinase (signal transducers and activators of transcription signaling pathways which acts with a dual role in inflammatory and autoimmune diseases [19–21]). It is seen that IL-22 leads to tumor proliferation, apoptosis suppression and metastasis promotion by activation of STAT3 in colon cancer [22]. Reversely, IL-22 exerts a protective role in mucosal wound healing acceleration by inducing STAT3-dependent expression in ulcerative colitis [23, 24].

To the best of our knowledge, no previous study has shown data that considers Th22 cells and their association with Th17 or Th1 in cervical cancer. To examine the possible status of these cells in the pathophysiology of CC, we measured the frequency of peripheral Th22, Th17, Th1, mRNA expression levels of RORC, AHR, IL-6, TNF-α in PBMCs along with plasma concentrations of IL-22, IL-17 and TNF-α in PB of CC, CIN patients and HC for assessing their relevance.

## Methods

### Patients and controls

Six-one pathologically confirmed CC patients (age 24 –60 years, median 48 years) and 38 CIN patients (age 27–61 years, median 42 years) were enrolled in this study. All the patients of CIN group have biopsy results of CINIII. Before the study none of the patients had received anticancer treatment.

Thirty-two healthy women with normal results of pap smear (TCT) and HPV (HC2) tests served as controls (age 22–47 years, median 27 years).They are from our Gynecologic Clinic and Regular Physical Examination Center.

The participants with simultaneous active or chronic infection, autoimmune disease, diabetes, or a history of other malignant tumors or connective tissue diseases were excluded. The characteristics of the patients are given in Table 1. The clinical stage of CC patients was based on FIGO 2009 criteria. Informed written consent was obtained from each participant. Medical Ethical Committee of Qilu Hospital, Shandong University, China provided the ethical approval for the study.

**Table 1:** Clinical characteristics of CC patients

Characteristics	Category	*N* = 61(%)
FIGO stage		
	IA	10 (16)
	IB	37 (61)
	IIA	9 (15)
	IIB	5 (8)
Histology type		
	SCC	54(88)
	ADC	4(7)
	Unknown	3(5)
Tumor differentiation		
	Well	9(15)
	Moderate	11(18)
	Poor	33(54)
	Unknown	8(13)
Lymph node metastases		
	Positive	11(18)
	Negative	48(79)
	Unknown	2(3)
Tumor size(cm)		
	<4	43(70)
	≥4	18(30)
Vasoinvasion		
	Yes	12(20)
	No	44(72)
	Unknown	5(8)

Abbreviation: FIGO, International Federation of Gynecologists and Obstetricians; SCC,

squamous cell carcinoma; ADC, adenocarcinoma.

### Flow Cytometric Analysis

Intracellular cytokines were evaluated by flow cytometry to identify the cytokine-producing cells. Briefly, heparinized peripheral whole blood (200 μl) with an equal volume of Roswell Park Memorial Institute (RPMI) 1640 medium (Sigma Chemical, St Louis, MO, USA) was incubated for 4 h at 37 °C and 5 % CO2 in the presence of 25 ng/ml of phorbolmyristate acetate (PMA), 1 μg/ml of ionomycin and 1.7 μg/ml of Monensin (all from Alexis Biochemicals, San Diego, CA, USA). After incubation, the cells were stained with anti-CD4-PE-Cy5 monoclonal antibodies at room temperature (RT) in the dark for 15 minutes to delimitate CD4^+^ T cells. Then, to fix the cells, 100 μl Reagent A (FIX &PERM Kit, MultiSciences Biotech Co., Ltd.) was added to each sample at RT in the dark for 15 min. After washing the cells with 3 ml PBS, 100 μl of Reagent B (FIX &PERM Kit, MultiSciences Biotech Co., Ltd.) and the recommended dose of anti-IL17A-PE and anti-IL22-APC and anti-IFNγ-FITC monoclonal antibody were added to each sample after fixation and permeabilization. Samples were then incubated at RT in the dark for 15 min to stain IL-17, IL-22 and IFN-γ. Isotype controls were used to correct compensation and confirm antibody specificity. After washing the cells with 3 ml PBS, we added 300 μl PBS to re-suspend the cells for cytometric analysis. Stained cells were analyzed by flow cytometric analysis using a FACS cytometer equipped with Cell Quest software (BD Bioscience Pharmingen). All antibodies mentioned above were from eBioscience (San Diego, CA, USA). Th17, Th22 and Th1 cells are defined as CD4^+^IFNγˉIL17^+^IL22ˉ, CD4^+^IFNγˉIL17ˉIL22^+^ and CD4^+^IFNγ^+^ cells respectively.

### Quantitative real-time PCR analysis

Trizol (Invitrogen, America) was used for isolation of Total RNA from PBMCs. For reverse transcription reaction the Prime Script RT reagent kit (Perfect Real Time; Takara) was used according to the instruction of the manufacturer. Reverse transcription reaction was done at 37 °C for 15 minutes, followed by 85 °C for 5 seconds. Real-time PCR was done by Applied Biosystems 7500 Real-Time PCR System (Applied Biosystems, Foster City, CA, USA). The primers are shown as follows: AHR forward: CAA ATC CTT CCA AGC GGC ATA; reverse: CGC TGA GCC TAA GAA CTG AAA G; RORC forward: TTT TCC GAG GAT GAG ATT GC; reverse: CTT TCC ACA TGC TGG CTA CA; TNF-α forward: CGA GTG ACA AGC CTG TAG C, reverse: GGT GTG GGT GAG GAG CAC AT; GAPDH forward: GCT CTC TGC TCC TCC TGT TC, reverse: GTT GAC TCC GAC CTT CAC CT; IL-6 forward: TTC TCC ACA AGC GCC TTC GGT CCA, reverse: AGG GCT GAG ATG CCG TCG AGG ATG TA. All experiments were conducted in triplicate. For calculation of amplification efficiency of the PCR products Applied Biosystems System software was used. The results were signified relative to the number of GAPDH transcripts used as a reference control.

### IL-22, IL-17 and TNF-α Enzyme-linked Immunosorbent Assay (ELISA)

Heparin-anticoagulant vacutainer tubes were used for collection of PB. For cytokines determination plasma was attained from all the subjects by centrifugation and stored at a temperature of −80 °C. A quantitative sandwich enzyme immunoassay technique was used for plasma level determination of IL-22, IL-17 and TNF-α according to the manufacturer’s instructions (eBioscience, San Diego, CA, USA).

### Statistical analysis

Mean ± SD or median (range) were used for expression of values. Distribution of the data was obtained from Kolmogorov-Smirnov test (K-S test). ANOVA and Newman-Kuels multiple comparison tests were used for the assessment of normal distribution data. Kruskal-Wallis test (H test) and Nemenyi tests were used for unusual data. Assessment of Correlation analysis was obtained from Pearson correlation. A *p* value less than 0.5 was considered statistically significant. All tests were performed by SPSS 17.0 software.

## Results

### Elevated Th22 and Th17 cells in PB of CIN and CC patients

The percentage of Th22 cells (CD4^+^IFNγˉIL17ˉIL22^+^ T cells, pure Th22 cells) and Th17 cells (CD4^+^IFNγˉIL17^+^IL22ˉ T cells, pure Th17 cells) of CIN (Th22: 1.27 ± 0.56 %, *p* = 0.001; Th17: 3.10 ± 1.40 %, *p* < 0.001) and CC patients (Th22: 1.75 % ± 0.704 %, *p* < 0.001; Th17: 3.35 ± 1.34, *p* < 0.001) significantly increased compared with HC (Th22: 0.77 % ± 0.36 %; Th17: 1.78 ± 0.80 % ). Besides, significant difference was also found in Th22 cells between CIN and CC patients (*p* < 0.001), but none in Th17 cells (Fig. 2a and b).

### Elevated Th1 cells in PB of CC patients

Significantly elevated frequencies of Th1 cells were found in CC (7.95 % ± 3.95 %) compared with HC (4.98 % ± 2.92 %, *p* < 0.001) and CIN patients (6.23 % ± 2.52 %, *p =* 0.015). However, no significant difference was found between HC and CIN patients (*p* > 0.05) (Fig. 2c).

**Fig. 1:**
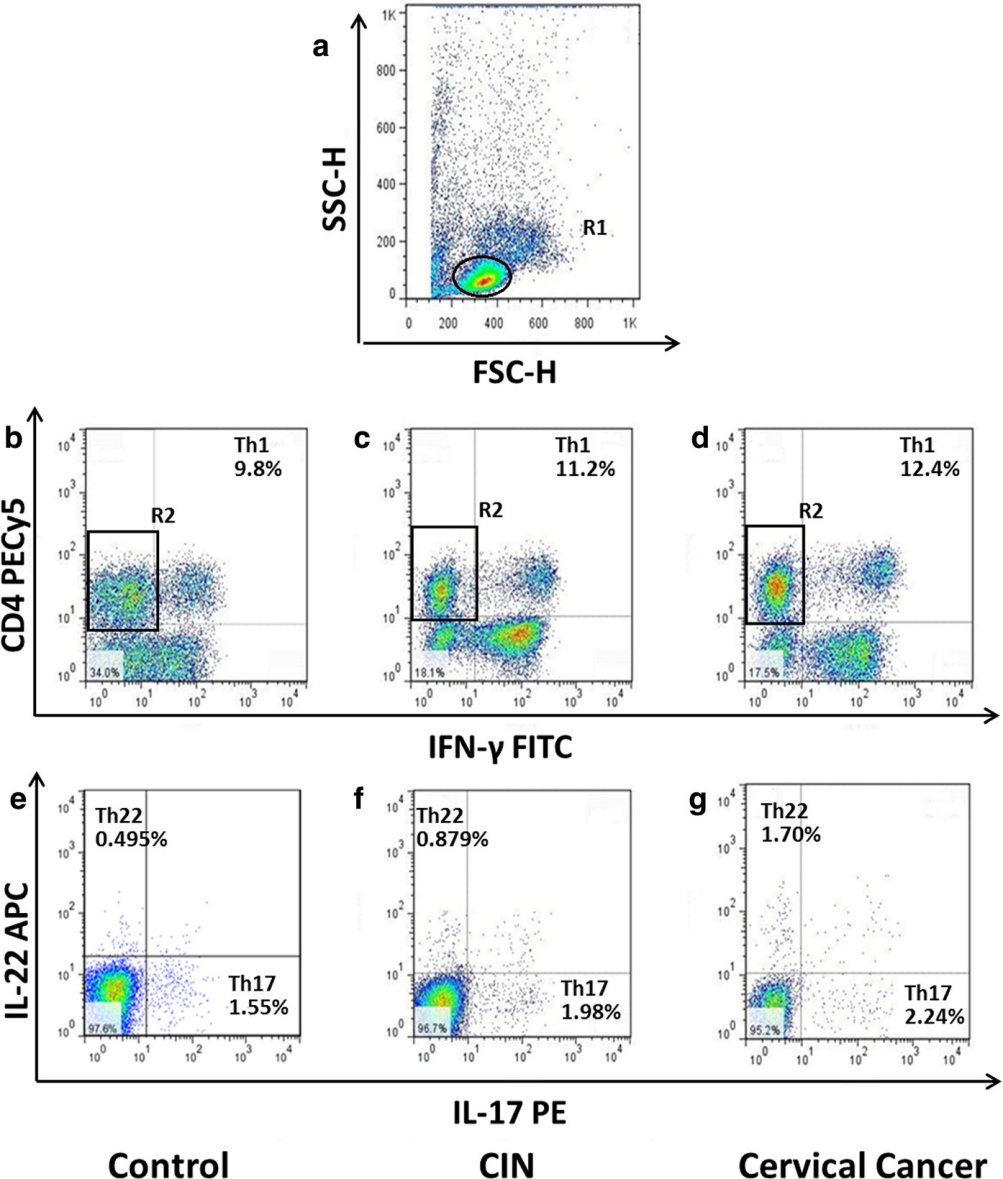
Circulating percentages of Th17, Th22 and Th1 cells in representative HC, CIN and CC patients. **a**. Lymphocytes were gated in R1 by flow cytometry. **b**, **c**, **d** The percentages of circulating Th1(CD4^+^ IFNγ^+^ T cells) cells in HC and CIN and CC patients. CD4^+^IFNγˉ T cells were gated in R2. **e**, **f**, **g** The proportions of pure Th17 (CD4^+^IFNγˉIL17^+^IL22ˉ T cells) and pure Th22 cells (CD4^+^IFNγ^−^IL17ˉIL22^+^ T cells) in representative controls, CIN and CC patients

A typical dot plot of the percentage of Th1 cells, Th22 cells and Th17 cells in representative patients and HC is shown in Fig. 1.

### Comparisons of Th17/Th22 ratio

**Fig. 2:**
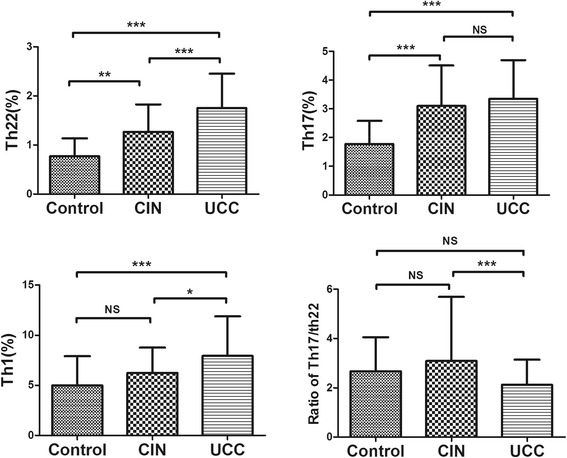
Results of circulating Th subsets in HC, CIN and CC patients. **a** The percentages of circulating Th22 (CD4^+^IFNγ^−^IL17ˉIL22^+^ T cells) cells. Significantly higher percentage of Th22 cells was present in CC patients (1.75 ± 0.704 %) in comparison with CIN patients (1.27 ± 0.56 %, *p* < 0.001) and HC (0.77 ± 0.36 %, *p* < 0.001); again increased percentage of Th22 cells noticed in CIN patients than HC (*p* = 0.001). **b** The percentages of circulating pure Th17 (CD4^+^IFNγˉIL17^+^IL22ˉ T cells) cells. There was a significantly high percentage of pure Th17 cells in CIN patients (3.10 ± 1.40 %, *p* < 0.001) or CC patients (3.35 ± 1.34 %, *p* < 0.001) than HC (1.78 ± 0.80 %). **c** The percentages of circulating Th1 (CD4^+^IFNγ^+^ T cells) cells. Significantly elevated frequencies of Th1 cells were found in CC (7.95 % ± 3.95 %) compared with HC (4.98 % ± 2.92 %, *p <* 0.001) and CIN patients (6.23 % ± 2.52 %, *p <* 0.001). However, no significant difference was found between HC and CIN patients. **d** Correlation of Th17/Th22 ratio in HC, CIN and CC patients. Significant difference was found between CIN (3.09 ± 2.60) and CC patients (2.12 ± 1.02, *p* = 0.007). Bars symbolized SD. * *p* < 0.05, ** *p* < 0.01, *** *p* < 0.001. NS no significance

Regarding the ratio of Th17/Th22, we detected a significant decrease in CC patients (2.12 ± 1.02, *p* = 0.007) compared with CIN patients (3.09 ± 2.60) (Fig. 2d).

### Correlation Analysis among Th22, Th17 and Th1 Cells in CC and CIN patients

**Fig. 3:**
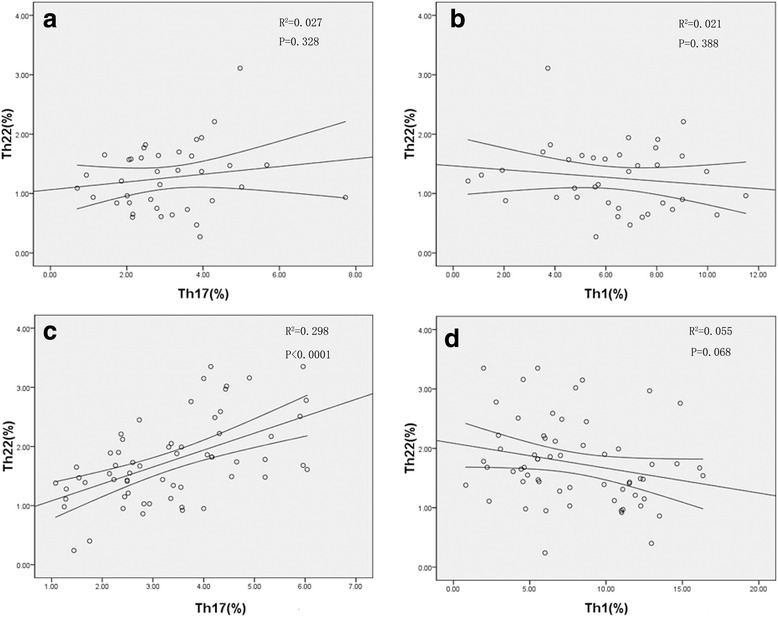
Correlations between Th subsets in CIN and CC patients. **a** The correlation between the levels of Th17 and Th22 cells in patients with CIN (r = 0.163, *p* = 0.328); **b** The correlation between the levels of Th22 and Th1 cells in patients with CIN (r = − 0.144, *p* = 0.388); **c** There was a positive correlation between Th22 cells and Th17 cells in CC patients (r = 0.546, *p* < 0.0001) **d** There was an approximately negative correlation between Th22 cells and Th1 cells in CC patients (r = − 0.235, *p* = 0.068).

A positive correlation was discovered among Th22 cells and Th17 cells in CC patients (r = 0.546, *p* < 0.0001, Pearson correlation analysis), but none in CIN patients (r = 0.163, *p* = 0.328). In CC patients, an approximately negative correlation was seen among Th22 and Th1 cells (r = − 0.235, *p* = 0.068, Pearson correlation analysis), but none in CIN patients (r = − 0.144, *p* = 0.388) (Fig. 3).

### mRNA expression levers of AHR, RORC, TNF-α and IL-6 in CC, CIN patients and controls

**Fig. 4:**
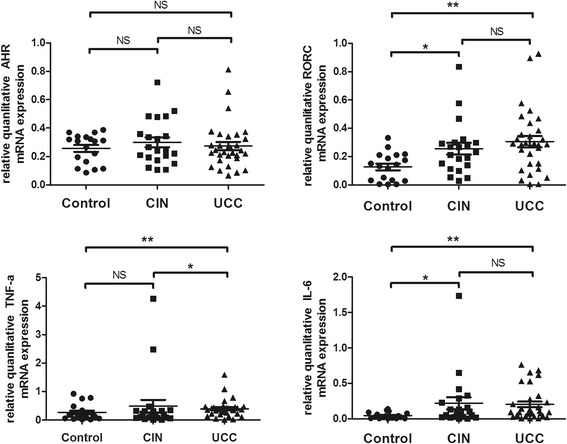
The mRNA expression of AHR, RORC, TNF-α and IL-6 in CIN and CC patients and HC. **a** AHR mRNA expression level between CIN patients, CC patients and HC was comparable (*p* > 0.05); **b** A remarkably high expression of the RORC mRNA was seen in CC patients (0.305 ± 0.188, *p* = 0.002) or CIN patients (0.256 ± 0.188, *p* = 0.036) compared to HC; **c** A significantly high expression of TNF-α was observed in CC patients (median, 0.369; range, 0.016 - 1.59) compared to CIN patients (median, 0.193; range, 0.009 - 4.27, *p* = 0.015) or HC (0.264 ± 0.28, *p* = 0.043); **d** The expression of IL-6 is significantly increased in CC patients (median, 0.101; range, 0.006 - 0.763, *p* = 0.001) or CIN patients (median, 0.085; range, 0.003 - 1.74, *p* = 0.019) when compared with HC (median, 0.029; range, 0.002 - 0.139). Bars symbolize SD. * *p* < 0.05, ** *p* < 0.01 NS no significance

There was an increased trend of AHR in CC patients (0.274 ± 0.160) and CIN patients (0.299 ± 0.16) compared with HC (0.257 ± 0.103), though both values of *P* were more than 0.05 (Fig. 4a).

**Fig. 5:**
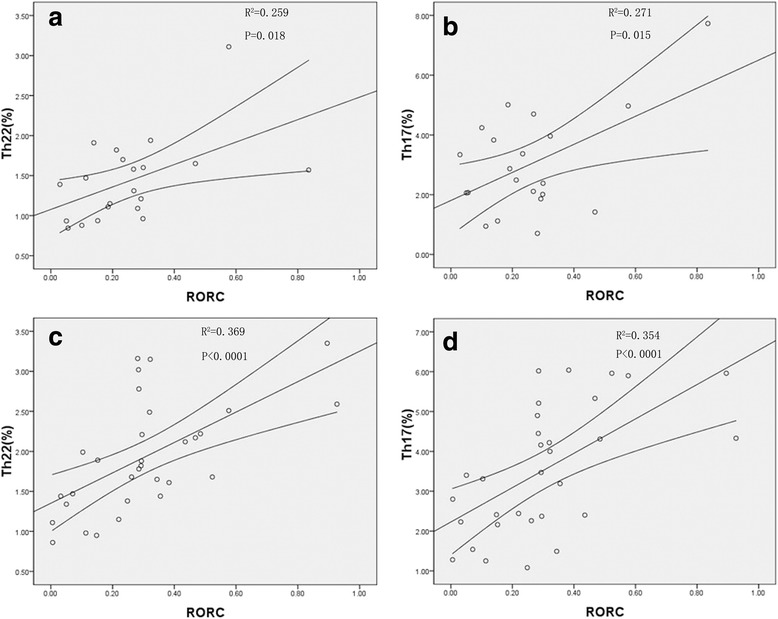
Correlations between RORC and Th subsets in CIN and CC patients. **a, b** RORC had the positive correlation with Th17 cells and Th22 cells in CIN patients (Th17 cells, r = 0.521, *p* = 0.015, *n* = 21; Th22 cells, r = 0.509, *p* = 0.018, *n* = 21); **c, d** RORC had the positive correlation with Th17 cells and Th22 cells in CC patients (Th17 cells, r = 0.600, *p* < 0.01, *n* = 31; Th22 cells, r = 0.612, *p* < 0.01, *n* = 31)

In comparison, CC patients (0.305 ± 0.188, *p* = 0.002) or CIN patients (0.256 ± 0.188, *p* = 0.036) exhibited increased level of the RORC mRNA expression than normal controls (0.128 ± 0.099) but the CIN patients and CC patients had no important difference in between (*p* > 0.05) (Fig. 4b). In addition, CC patients (r = 0.60, *p* < 0.01, Pearson correlation) and CIN patients (r = 0.521, *p* = 0.015, Pearson correlation) had a positive correlation between RORC and Th17 cells. Furthermore, CC patients (r = 0.612, *p* < 0.01, Pearson correlation) and CIN patients (r = 0.509, *p* = 0.018, Pearson correlation) showed a positive correlation between RORC and Th22 cells (Fig. 5).

The CC patients (median, 0.369; range, 0.016 – 1.59) showed TNF-α mRNA expression significantly high in comparison with HC (0.264 ± 0.28, *p* = 0.043) and CIN patients (median, 0.193; range, 0.009 – 4.27, *p* = 0.015) but CIN patients and HC did not show any significant high level of this expression (Fig. 4c).

The HC (median, 0.029; range, 0.002 -- 0.139) had lower IL-6 mRNA expression in PBMCs than the CC patients (median, 0.101; range, 0.006 – 0.763, *p* = 0.001) and CIN patients (median, 0.085; range, 0.003 – 1.74, *p* = 0.019) but CIN patients and CC patients had no significant difference in between (*p* > 0.05) (Fig. 4).

### Correlation on the frequencies of Th17 and Th22 cells with clinical characters in CC patients

**Fig. 6:**
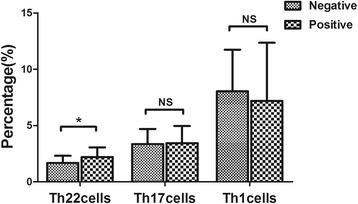
The Th22, Th17 or Th1 cells frequency in positive or negative lymph node metastases. Increased frequency (*p* = 0.026) of Th22 was observed in CC patients with lymph node metastases (2.20 ± 0.85 %, *n* = 11) comparing to CC patients without lymph node metastases (1.68 ± 0.64 %, *n* = 48). *p < 0.05, NS no significance

CC patients with lymph node metastasis exhibited profoundly increased frequency of Th22 cells (2.20 ± 0.85 %, *n* = 11) compared to CC patients without lymph node metastases (1.68 ± 0.64 %, *p* = 0.026, n = 48) (Fig. 6). No significant diversity was detected among Th22, Th17 and Th1 cells frequency and other prognostic factors including clinical stage, tumor size and vasoinvasion in CC patients (*p* > 0.05).

### Increased IL-22 concentrations in plasma of CC patients

**Fig. 7:**
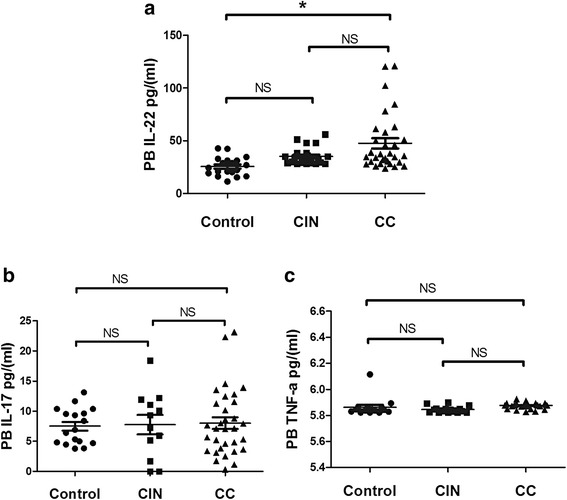
Results of plasma cytokines in CIN, CC patients and HC. **a** A significantly elevated expression of IL-22 was seen among CC patients (median 37.46; range 24.84 - 120.06 pg/ml, *p* = 0.039) and HC (median 26.8;range 11.3-42.7 pg/ml). **b** No significant difference was found on concentration of IL-17 in control, CIN and CC patients. **c** No significant elevation was found on concentration of TNF-α in control, CIN and CC patients. *p < 0.05 NS no significance

The CIN or CC patients and HC all showed plasma IL-22, IL17 and TNF-α. Significantly higher levels of IL-22 were revealed in CC patients (median 37.46; range 24.84 – 120.06 pg/ml, *n* = 31, *p* = 0.039) than those in HC (median 26.8; range 11.3-42.7 pg/ml, n = 19) (Fig. 7a). No remarkable diversities were found among CIN patients (CIN: median 31.17; range 20.93 - 82.68 pg/ml, *n* = 22, *p* > 0.05) and CC patients or CIN patients and HC.

However, concentration of plasma IL-17 and TNF-α were found similar in HC, CIN and CC patients (*p* > 0.05) (Fig. 7b and c).

## Discussion

Persistent infection with HPV is the main cause of CC and CIN [25, 26]. That CIN and CC arise more frequently in immunosuppressive women indicates that elimination of HPV is related to immunity function. In the evolution of these diseases, local or systemic immune mechanisms abnormalities may be involved [27, 28]. A vast and dynamic crosstalk among immune cells, along with cytokines turmoil has been regarded as a crucial element of cancer pathophysiology [29]. In our current study, we focused on immune cells, mainly three subtypes of T helper cells- Th1, Th17 and Th22 cells and their probable role in CC and CIN.

Interferon (IFN)-γ causes activation of immune cells in the tumor microenvironment. It is known that Th1 cells, the main source of IFN-γ, have a powerful anti-tumor function. To enhance the function of antigen presenting cells, tumor antigen specific CD4^+^Th1 cells can travel to the tumor site and secrete inflammatory cytokines and modulate the microenvironment [30]. It was observed that for cancer inhibition and better outcomes, Th1 adaptive immunity is essential [31]. In our study, we demonstrated a significant elevated frequency of Th1 cells in CC patients, compared to CIN patients and HC, which is consistent with other previous studies of the involvement of Th1 cells in tumors.

It was noticed that another two inflammatory cell subgroups, Th17 and Th22 cells are involved in viral infection and mucosal immunity [32, 33, 34]. In our previous study, we saw that there was a significant increase of Th17 cells (CD4^+^IL17^+^ cells) in CIN and CC patients [8, 11]. In order to exclude multiple positive cells Th17 cells are defined as CD4^+^IFNγ^−^IL17^+^IL22^−^ cells, which also were called as “pure Th17 cells”. Th22 cells are now defined as CD4^+^IL17ˉIL22^+^IFNγ^−^ cells, which is an independent subset of T helper cells from Th1 and Th17 cells [35–37]. In the current study, we evaluate the frequencies of pure Th17 and Th22 cells to confirm the probable role of these two famous types of T helper cells in PB of CIN and CC by flow cytometry. As expected, increased frequencies of Th17 and Th22 cells were found in both CIN and CC compared to HC. Moreover, the increased change of Th22 cells in CC was much higher than that of CIN. It suggested that as cervical precancerous lesion occurs, Th22 cells might gradually elevate from CIN to CC. However, no significant difference of Th17 cells was found between CIN and CC. But the data indeed shows that there are frequencies of Th17 and Th22 cells changed in the tumorigenesis of both CIN and CC which indicate these two types of cells may paticipate in tumor immunity.

IL-22 is known to have a relationship with virus-infection reactions and whose receptor is confined to non-hematopoietic cells (mainly epithelial cells). Previously it was considered that IL-22 is a cytokine of Th17 cells. Now it is considered as the characteristic product of Th22 cells. Our study also revealed elevated levels of plasma IL-22 in CC patients. Additionally, expression of a series of molecules, which are responsible for cellular differentiation and survival was triggered by IL-22 [38, 39]. In our study, a raised level of plasma IL-22 was found, which indicated that Th22 cells, the main T helper cells which product IL-22, may be involved in the process of CC. However, plasma IL-17 did not show a significant change. This might be due to the fact that concentration of IL-17 was too low to present the change, as it showed low levels in both of CC and HC. In addition, there was a positive correlation between the frequencies of Th17 and Th22 cells in CC patients, suggesting that differentiation of Th22 cells may be linked to Th17 cells or even Th22 cells might partly derive from Th17 cells. This derivation may partly explain the type of IL22^+^Th17 cells. However, no correlation was found in CIN III or HC. One reason for this is that the frequencies level of Th17 and Th22 cells are very low, hence the difference between detected results and real conditions multiplied and distorted the statistic results. Another reason is that, in a normal situation, Th17 and Th22 cells are derived from a different origin and induced by different stimuli. However, when cancer appears, inflammatory cells show a partly inter-related differentiation, which also causes elevated frequency of IL22^+^Th17 cells during the process.

It was seen that RORC is the key transcription factor directing Th17 lineage and modulates the polarization of Th22 cells [12, 40]. In our study we noticed a notably elevated expression of RORC in CIN and CC patients. Also, the expression of RORC is positively correlated with both Th22 and Th17 cells. It is assumed that in CIN and CC patients the differentiation of Th22 and Th17 cells is mainly regulated by RORC. We previously found that IL-6, which promoted differentiation of Th22 cells, is highly expressed in CIN and CC patients [11, 12, 19]. Elevated IL-6 mRNA expression was found in CIN and CC patients compared to HC. The data showed that, in CC and CIN patients, immune environment may be more suitable for polarization of Th22 cells.

However, no significance was found in AHR expression. Although AHR is the most important transcription factor of Th22 cells, AHR pathway is not unique. It is demonstrated that TGF-β could inhibit IL-22 secreting of Th17 cells by AHR-independent pathways. In our study of CIN and CC, no significant change was found. The explanation for increase of Th22 cells may not be caused by AHR (transcription level), but others pathways, such like stimulation and transformation.

Referring to clinic factors, in CC patients, lymph node metastases were found to correlate with aggregation of Th22 cells. Again, a positive association between Th22 cells and Th17 cells was also observed. Consequently, it is imaginable that co-increased levels of Th22 and Th17 cells along with pro-inflammatory cytokines may play a synergistic role in the progression of CC. Nevertheless, there was an approximately negative correlation between Th1 cells and Th22 cells in CC patients. This argues that the beneficial Th1 cells gradually declined while more Th22 was produced toward disease progression. However, the interaction among these three different cells demands further investigation.

## Conclusion

It is seen that patients with CC possess a high frequency of circulating Th22 cells, Th17 cells and Th1 cells. The higher prevalence of Th22 cells was found in patients with advanced CC, arguing an important role for this T-cell subtype in the growth and acceleration of CC. For a better understanding of this development (i.e., regulation and function of these cells in CC) more extensive experiments are needed which may lead to the evolution of promising therapeutic strategy for CC patients.

## Abbreviations

AHR: Aryl hydrocarbon receptor
CC: Cervical cancer
CIN: Cervical intraepithelial neoplasia
HC: Healthy control
HPV: Human papilloma virus
PB: Peripheral blood
PBMCs: Peripheral blood mononuclear cells
PCR: polymerase chain reaction
RORC: RAR-related orphan receptor C
SD: Standard deviation
STAT: signal transducer and activator of transcription
Th: T helper cell
TNF: Tumor necrosis factor
Treg: The regulatory T cells
